# Comprehensive Analysis of Chemotherapeutic Agents That Induce Infectious Neutropenia

**DOI:** 10.3390/ph14070681

**Published:** 2021-07-15

**Authors:** Mashiro Okunaka, Daisuke Kano, Reiko Matsui, Toshikatsu Kawasaki, Yoshihiro Uesawa

**Affiliations:** 1 Department of Medical Molecular Informatics, Meiji Pharmaceutical University, Kiyose, Tokyo 204-8588, Japan; mokunaka@east.ncc.go.jp; 2 Department of Pharmacy, National Cancer Center Hospital East, Kashiwa, Chiba 277-8577, Japan; dkano@east.ncc.go.jp (D.K.); rmatsui@east.ncc.go.jp (R.M.); tkawasak@east.ncc.go.jp (T.K.)

**Keywords:** chemotherapy-induced neutropenia, chemotherapeutic agent, cancer

## Abstract

Chemotherapy-induced neutropenia (CIN) has been associated with a risk of infections and chemotherapy dose reductions and delays. The chemotherapy regimen remains one of the primary determinants of the risk of neutropenia, with some regimens being more myelotoxic than others. Although a number of clinical trials have currently highlighted the risk of CIN with each chemotherapy regimen, only a few ones have comprehensively examined the risk associated with all chemotherapeutic agents. Therefore, this study aimed to investigate the risk factors and characteristics of CIN caused by each neoplastic agent using data from the large voluntary reporting Food and Drug Administration Adverse Event Reporting System database. Initially, univariate analysis showed that an age ≥ 65 years, the female sex, and treatment with chemotherapeutic agents were factors that caused CIN. Then, cluster and component analyses showed that cytotoxic agents (i.e., alkylating agents, antimetabolic agents, antineoplastic antibiotics, platinating agents, and plant-derived alkaloids) were associated with infection following neutropenia. This comprehensive analysis comparing CIN risk suggests that elderly or underweight patients treated with cytotoxic drugs require particularly careful monitoring.

## 1. Introduction

Neutrophils generally comprise approximately half to two-thirds of all white blood cells (immune cells) and protect against bacterial infections [1]. Patients who develop neutropenia may have a higher-than-normal risk of infections, and the severity of subsequent infections is also higher. Chemotherapeutic agents act on the bone marrow, where active cell division occurs, and deplete hematopoietic stem cells [2], leading to a decreased circulating absolute neutrophil count [3]. Patients receiving chemotherapy have been reported to experience a temporary reduction in their neutrophil counts [4,5]. Chemotherapy-induced neutropenia (CIN) remains a common dose-limiting toxicity for chemotherapeutic agents, causing treatment delays and/or dose reductions [6].

Furthermore, CIN increases in severity as the absolute neutrophil count declines below 500/μL [5]. Febrile neutropenia refers to the occurrence of fever during a period of severe neutropenia.

Although a number of clinical trials have investigated the risk of CIN across multiple chemotherapy regimens, comparing their results based on indications remains challenging. Furthermore, triplet/doublet regimens promote a higher CIN incidence and myelotoxicity than single-agent regimens [7]. A retrospective comparative study on the toxicity of multiple chemotherapeutic agents frequently observed febrile neutropenia with paclitaxel for breast cancer (18%), carboplatin plus paclitaxel for lung cancer (23%), and oxaliplatin and fluorouracil plus leucovorin for colorectal cancer (23%) [8]. However, only a few studies have comprehensively examined the risk associated with multiple chemotherapeutic agents, and to the best of our knowledge, none have comprehensively investigated all agents.

The US Food and Drug Administration (FDA) publishes the FDA Adverse Event Reporting System (FAERS) database. This is a voluntary reporting system database for the postmarketing surveillance of all approved drugs and therapeutic biologics, available to public and scientists from the FDA’s home page [9,10]. The present study comprehensively analyzed all chemotherapeutic agents available in the FAERS database and compared CIN risk and detailed characteristics.

## 2. Results

### 2.1. Data Presentation

**Figure 1:**
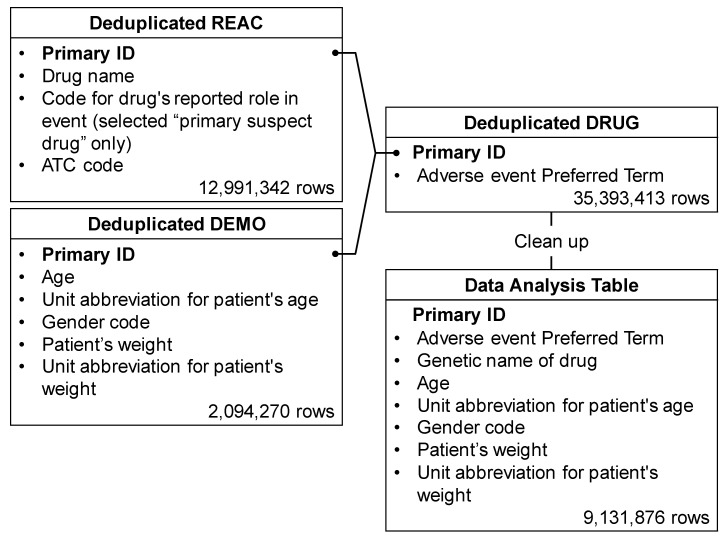
Flow chart for the construction of the data analysis table. The REAC table was classified into three categories: “suspected medicine”, “concomitant medicine” and “interaction medicine”. We extracted only “primary suspect drug” information relevant to these categories from the REAC table. Duplicate data were then removed from the REAC, DEMO, and DRUG tables. Based on the combined table, only “suspected medicine” information was used to assess the risk of diarrhea. Available information on “suspected medicine”, “concomitant medicine” and “interaction medicine” was used for the time-of-onset analysis. Each table was combined, cleaned up, and then used as the data analysis table.

Among the 35,393,413 rows (21,349 categories) of adverse events registered in the FAERS, 121,722 rows (eight categories) were related to neutropenia. The drug/biologic information for any medication as associated with an event (DRUG), MedDRA terms coded for adverse events (REAC), and patient demographic and administrative information (DEMO) tables included 35,393,413, 12,991,342, and 2,094,270 rows, respectively. The total number of rows, as shown in the data analysis table, was 9,131,876 (Figure 1).

### 2.2. Patients Characteristics

Significant differences in sex, age, and weight characteristics were observed in patients who developed neutropenia. Criteria for elderly patients aged ≥ 65 years were defined based on a previous study regarding risk factors for febrile neutropenia in patients with cancer who are receiving chemotherapy [11]. More females than males showed a tendency to develop CIN (51.7%, *n* = 16,751, vs. 48.3%, *n* = 15,640; Table 1 and Table 2). Patients with CIN had a median age and weight of 61 years and 68.0 kg, respectively, whereas those without CIN had a mean age and weight of 58 years and 73.0 kg, respectively.

### 2.3. CIN-Inducing Drugs

**Figure 2:**
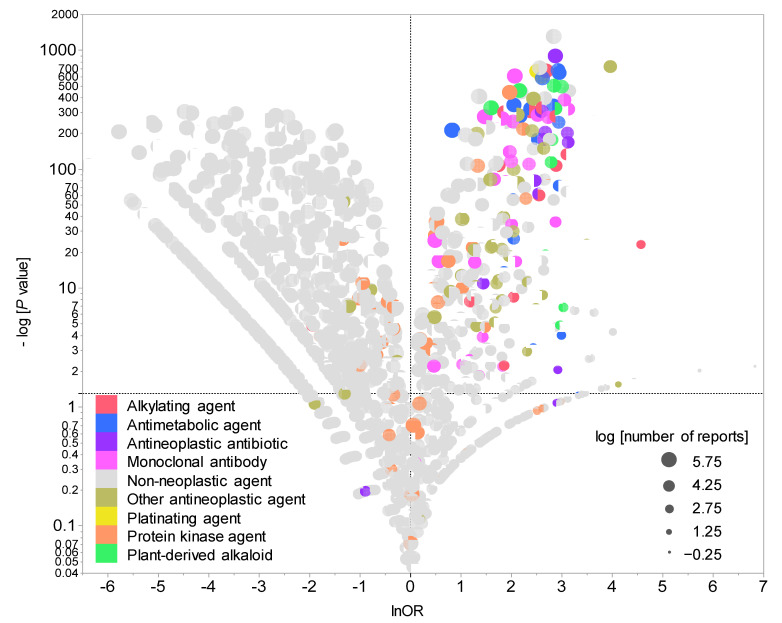
Drugs associated with CIN development. The X axis shows the natural lnORs, whereas the Y axis shows the common logarithm of the inverse *p* value (−log10(*p* value)) from Fisher’s exact test. The ORs were calculated using cross-tabulation. The dotted line on the Y axis represents *p* = 0.05. The plot colors indicate Anatomical Therapeutic Chemistry classification, whereas the plot size indicates the common logarithm of the total number of reported adverse events for each drug (−0.25 to 5.75).

Figure 2 presents a volcano plot demonstrating drugs suspected of causing neutropenia. As shown in the figure, drugs with positive lnORs on the X axis were more frequently reported to have caused neutropenia than other adverse events. Moreover, drugs with higher values of logarithmically transformed inverse *p* values on the Y axis had stronger significant differences. In other words, the drugs located in the right upper quadrant were more likely to induce medication-related neutropenia. The volcano plot showed that cytotoxic agents such as alkylating agents, antimetabolic agents, and antineoplastic antibiotics were particularly associated with the development of neutropenia.

### 2.4. Cluster Analyses of CIN-inducing Drugs

**Figure 3:**
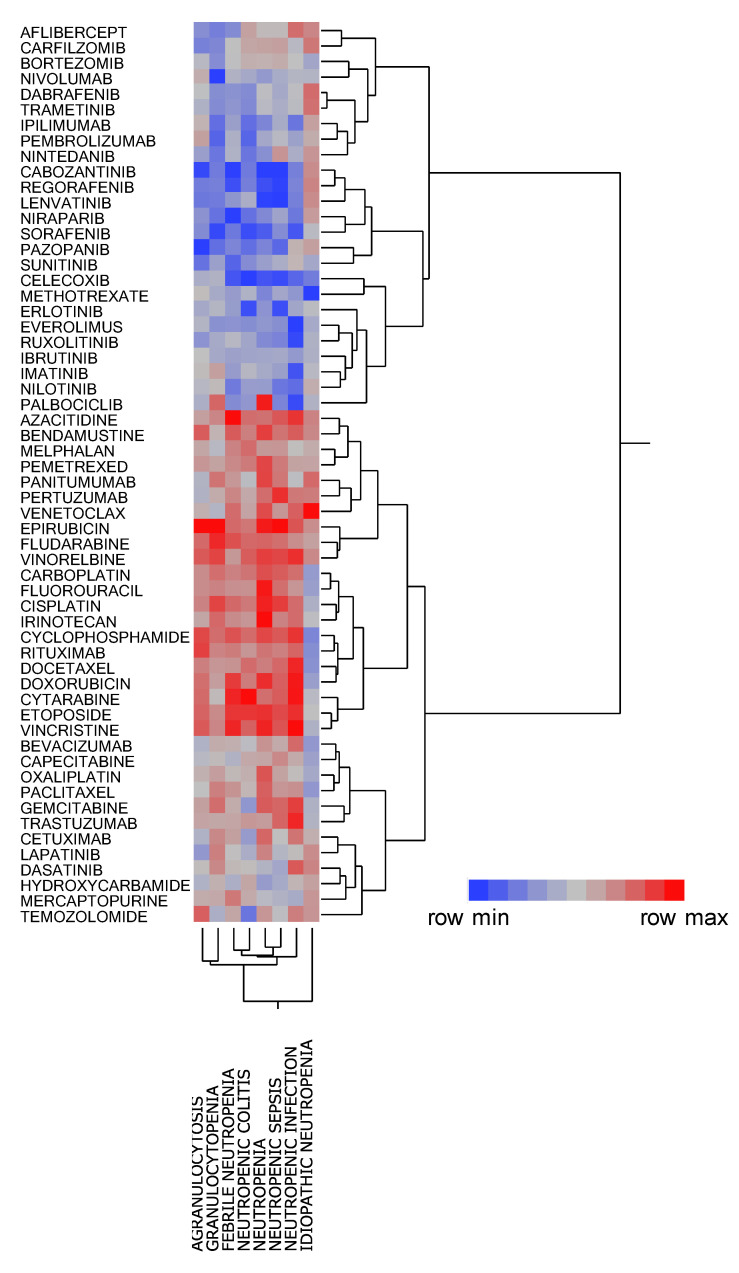
Classification of CIN-related chemotherapeutic agents using hierarchical cluster analysis. The dendrogram shows the relationships between 58 chemotherapeutic agents and CIN. The color map shows the load value of the principal components in red–gray–blue.

The dendrogram generated by hierarchical cluster analysis resulted in two clusters (Figure 3), among which one was associated with neutropenia, granulocytopenia, agranulocytosis, neutropenic sepsis, neutropenic colitis, neutropenic infection, and febrile neutropenia. This cluster contained the cytotoxic agents (e.g., epirubicin, vinorelbine, and cyclophosphamide) and monoclonal antibodies (e.g., bevacizumab and trastuzumab) that are administered in combination with cytotoxic agents. Other than palbociclib which was strongly associated with neutropenia and granulocytopenia, the second cluster contained drugs that were poorly associated with these adverse events, such as protein kinase agents (e.g., regorafenib and imatinib) and immune checkpoint inhibitors (e.g., nivolumab and ipilimumab). Thus, different degrees of association with CIN were noted for each chemotherapeutic agent.

### 2.5. Principal Component Analysis Related to CIN

**Figure 4:**
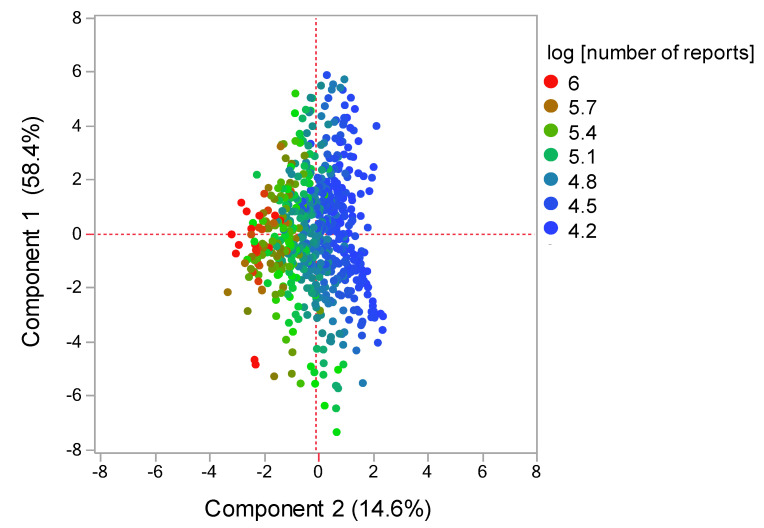
Score plot related with components 1 and 2. The score plot shows the relationships between the drugs and principal components. Each plot indicates a drug. Plot colors indicate log (number of reports).

**Figure 5:**
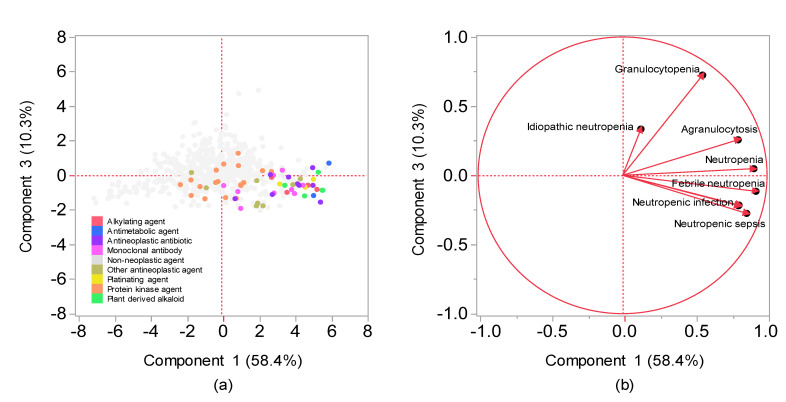
Relationships between CIN and drugs using principal component analysis. (**a**) Score plot; (**b**) loading vectors. (**a**) The score plot shows the relationships between the drugs and principal components. Each plot indicates a drug. Plot colors indicate Anatomical Therapeutic Chemistry classification. (**b**) Loading vectors represent the relationship between side effects and principal components. Each loading vector indicates a side effect.

The contribution ratios of the principal component were 58.4%, 14.6%, and 10.3% for the components 1, 2, and 3, respectively. Component 2 was excluded from the variable selection given its possible association with the number of reports (Figure 4). A scatterplot was then created using components 1 and 3. The relationship between adverse events related to neutropenia and main components was visualized using the plot, where each adverse event was represented as a loading vector (Figure 5). The X axis represents the first component, with all adverse event vectors showing a positive association. The Y axis represents the third component in which granulocytopenia (0.670), idiopathic neutropenia (0.383), agranulocytosis (0.242), and neutropenia (0.0118) showed a positive principal component load, whereas febrile neutropenia (−0.141), neutropenic colitis (−0.179), and neutropenic infection (−0.187), and neutropenic sepsis (−0.288) showed a negative principal component load (Table 3).

## 3. Discussion

The present study examined the risk factors and characteristics of CIN and clarified the relationship between CIN and related disease. To the best of our knowledge, this is the first study to provide data on the frequency of comprehensive CIN for all drugs using the FAERS database.

Our results showed that elderly patients, those receiving antineoplastic agents, and underweight individuals (low BMI/body surface area) were more likely to develop CIN (Table 1 and Table 2). In particular, alkylating agents (e.g., cyclophosphamide), plant-derived alkaloids (e.g., docetaxel), antineoplastic antibiotics (e.g., doxorubicin), and platinating agents (e.g., cisplatin) have been found to be myelosuppressive. Although it was difficult to calculate BMI because the FAERS database does not contain height information, the findings presented in this study, with the exception of sex, showed a tendency to be consistent with those reported in a previous study regarding risk factors for febrile neutropenia among patients with cancer who were receiving chemotherapy [11]. In other words, patients who present with CIN risk factors should also be vigilant regarding the development of febrile neutropenia following myelosuppression.

This study also investigated the relationship between CIN and chemotherapeutic agents. Cluster analysis, a method for classifying data into similar groups (clusters) [12], revealed that neutropenia, granulocytopenia, agranulocytosis, neutropenic sepsis, neutropenic colitis, neutropenic infection, and febrile neutropenia shared similar features (Figure 3). The present study classified 48 drugs into two clusters before evaluating the cluster characteristics. Notably, one cluster showed a strong positive association with neutropenia, granulocytopenia, agranulocytosis, neutropenic sepsis, neutropenic colitis, neutropenic infection, and febrile neutropenia, whereas the other, comprising protein kinase inhibitors and monoclonal antibodies (excluding the antimetabolite methotrexate), was less associated with CIN.

Our findings showed that palbociclib, a protein kinase inhibitor, was strongly associated with neutropenia and granulocytopenia but weakly associated with febrile neutropenia, neutropenic colitis, neutropenic sepsis, and neutropenic infection, with asymptomatic neutropenia being considered the main adverse effect of palbociclib. The PALOMA-3 trial showed that although 58–92% of patients had ≥grade 3 neutropenia, no complications of infection and increase in febrile neutropenia had occurred [13]. Palbociclib has been reported to exert its antitumor effects by inhibiting CDK4/6 [14], which is significant owing to evidence showing that the mechanism for neutropenia development may differ from that due to cytotoxic chemotherapy.

Although cluster analysis classifies drugs with varying characteristics based on the index of similarity, comprehensively comparing the bias of explanatory variables of drugs remains difficult. Principal component analysis has been used as a method for visualizing the bias of explanatory variables by converting them into a summary index (principal component) [15]. Each adverse event and drug used in the present study was interpreted based on principal components, whereas the main component was interpreted using loading vectors that represent adverse events (Figure 4 and Figure 5). Given that all adverse event vectors were positively associated with component 1, such a component can be considered a comprehensive index of CIN-related side effects. Adverse event vectors were classified into those with positive and negative relationships with components 2 and 3. In component 2, the positive adverse events included idiopathic neutropenia, neutropenic infection, and neutropenic colitis, whereas negative adverse events included febrile neutropenia, neutropenic sepsis, neutropenia, agranulocytosis, and granulocytopenia. However, Figure 4 shows that the positive–negative relationship in component 2 was associated with the number of reports. In component 3, the positive adverse events included granulocytopenia, idiopathic neutropenia, agranulocytosis, and neutropenia (Figure 5), whereas the negative adverse events included febrile neutropenia, neutropenic colitis, and neutropenic sepsis, which have been considered to affect bacterial infection. Therefore, component 3 can be considered a comprehensive indicator of infections following myelosuppression.

Drugs were also interpreted using a score plot (Figure 5). Most chemotherapeutic agents showed a positive association with both components 1 and 3, with nonchemotherapeutic agents showing a tendency to be a negatively associated with either the first or the second principal component. Among the antineoplastic drugs, cytotoxic agents (i.e., alkylating agents, antimetabolic agents, antineoplastic antibiotics, platinating agents, and plant-derived alkaloids) were found to be associated with infection following neutropenia. Monoclonal antibodies, which are strongly associated with infection following neutropenia, include drugs that are generally used in combination with cytotoxic agents (e.g., bevacizumab and trastuzumab) and drugs indicated for hematological malignancies (e.g., rituximab). Moreover, the effects of protein kinase inhibitors on neutrophils were plotted separately from cytotoxic agents. The results obtained from this study may help healthcare professionals to appropriately manage drug-induced adverse effects in patients.

### Limitations

This study has three limitations [16,17]. First, although mild adverse effects were only occasionally reported, severe adverse effects were frequently reported, which can lead to reporting bias, a characteristic of self-reporting databases [18]. Second, data obtained from the FAERS database contain blank cells, with some reports having incorrect characters and numbers. Therefore, this study needed to revise the side effects and drug names as much as possible. Third, the cause of the side effects was difficult to determine when multiple drugs were administered [16,17].

## 4. Materials and Methods

### 4.1. Database Information

Since January 2004, the U.S. FDA has continued to add information regarding cases of adverse events associated with all marketed drugs and therapeutic biologic products to the FAERS database [10], which contains adverse events reported by manufacturers to the FDA as required by regulation, along with reports received directly from consumers and healthcare professionals. After downloading the FAERS database, analysis was performed using data reported between April 2004 and September 2020.

### 4.2. Terminology of Analyzed Drugs and Adverse Events

The analyzed drugs were selected from the FAERS database using the World Health Organization-recommended Anatomical Therapeutic Chemistry (ATC) classification system [19]. “L01: antineoplastic agents” (222 drugs) were extracted from the “ANTINEOPLASTIC AND IMMUNOMODULATING AGENTS” class, one of the 14 major ATC classes.

To analyze CIN, eight preferred terms (i.e., neutropenia (PT code: 10029354), idiopathic neutropenia (PT code: 10051645), granulocytopenia (PT code: 10018687), agranulocytosis (PT code: 10001507), neutropenic sepsis (PT code: 10049151), neutropenic colitis (PT code: 10062959), neutropenic infection (PT code: 10059482), and febrile neutropenia (PT code: 10016288)) were extracted based on MedDRA/J version 23.0.

### 4.3. Production of Data Analysis Table

**Figure 6:**
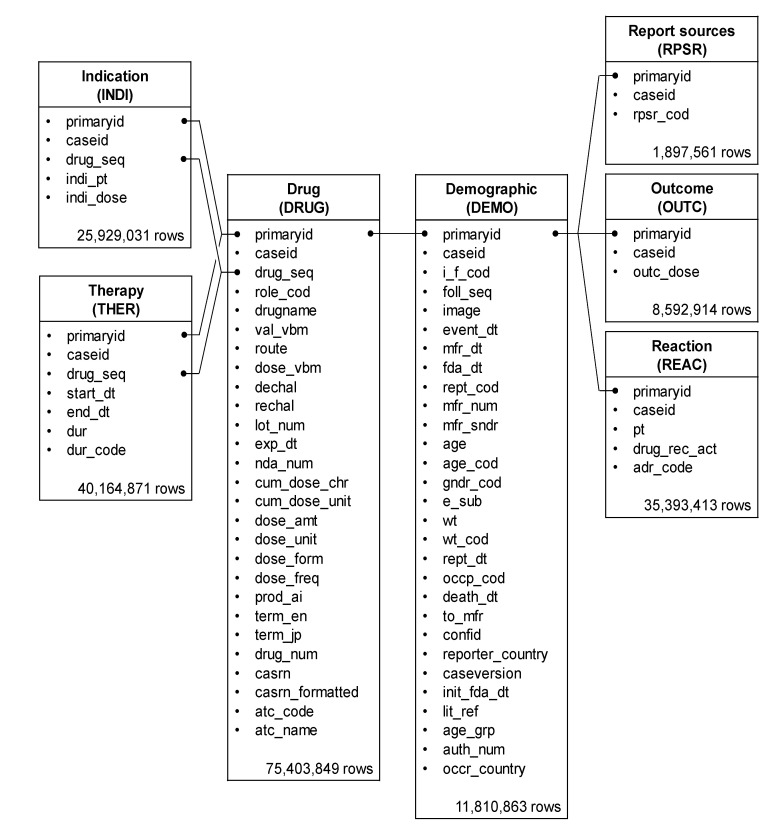
Seven information tables included in the FAERS database. The row number shows the number of reports obtained between April 2004 and September 2020.

The FAERS database comprises seven tables related to (a) (DEMO), (b) DRUG, (c) REAC, (d) patient outcomes for the event (OUTC), (e) report sources for the event (RPSR), (f) drug therapy start and end dates for the reported drug (THER), and (g) MedDRA terms coded for the indications (diagnoses) of the reported drugs (INDI). DRUG, REAC, and DEMO tables were analyzed to determine the patient’s background and drugs causing the neutropenia (Figure 6).

Duplicate data were removed from the DEMO, DRUG, and REAC tables and combined based on the primary ID. Furthermore, only information corresponding to the “primary suspect drug” was extracted from these data and used for constructing the data analysis table (Figure 1). Of this, pediatric patients with cancer, defined as patients with cancer or sarcoma aged < 15 years, accounted for 0.1% (2145 patients, 9336 rows).

### 4.4. Patient Characteristics Associated with Neutropenia

Patient characteristics were divided based on the presence and absence of CIN. Age and weight data from the data analysis table were treated as absolute numbers, and *p* values were calculated using Wilcoxon’s rank-sum test. Registered data for a weight of ≥150 kg was assumed to be 150 kg, whereas registered data for an age of ≥110 years was assumed to be 110 years. *p* values for sex were calculated using Fisher’s exact test. Patient factors were analyzed using only datasets that did not include missing values.

### 4.5. Univariate Analysis of Relationship between Drugs and Neutropenia

Reporting odds ratio (ROR) and Fisher’s exact test were used to assess the risk of CIN for each registered drug. Initially, a 2 × 2 contingency table of drugs and adverse events were created for each drug based on the information presented in the data analysis table (Table 4). Given that the 2 × 2 contingency table could not be calculated with zero cells and that the estimation would become unstable with a small cell frequency, 0.5 was added to all cells as a correction (Haldane Anscombe half correction) [20,21]. In this study, CIN-related drugs were defined as those with a ROR of ≥1 and a Fisher’s exact test *p* value of <0.05 [22]. Subsequently, a scatterplot comprising ROR and *p* values was created for the visual interpretation of adverse drug events. This scatter plot was created by converting ROR to logarithmically transformed odds ratios (lnORs) and the *p* value obtained from Fisher’s exact test to common logarithms (−log10(*p* value)). The scatter plot corresponds to volcano plots frequently used to understand gene expression trends in bioinformatics [23,24,25,26,27].

### 4.6. Cluster Analyses of CIN-Related Drugs

Among the 185 drugs identified as “L01: ANTINEOPLASTIC AGENTS”, 58 (31.4%) with ≥20,000 reports were analyzed. RORs were calculated from 2 × 2 contingency tables for adverse event and registered drugs. Thereafter, the RORs were converted to natural logarithms and used in hierarchical cluster analysis to objectively classify the registered drug with ≥20,000 reports. This analysis used the Ward method based on Euclidean distance with loads from 58 chemotherapeutic agents [12,28].

### 4.7. Principal Component Analysis Related to CIN

The cluster analysis analyzed and roughly classified the antineoplastic agents registered in FAERS (185 drugs, 6.0%) based on their characteristics. In contrast, the principal component analysis analyzed only antineoplastic agents with >20,000 reports (122 drugs, 4.0%) among all registered drugs (3079 drugs) in the data analysis table and compared more detailed CIN explanatory variable biases. RORs were calculated from 2 × 2 contingency tables for adverse events and registered drugs. Thereafter, the RORs were converted to natural logarithms and used in principal component analysis with association matrices [15,29]. The first, second, and third principal components were used to interpret the characteristics of the drugs and their adverse events.

### 4.8. Statistical Analysis

All analyses were performed using JMP Pro14 (SAS Institute Inc., Cary, NC, USA), with the level of statistical significance being set to 0.05.

## 5. Conclusions

Our comprehensive analyses of the data in a large dataset in order to compare the risk of CIN as well as the detailed characteristics of patients treated using chemotherapeutic agents highlight the necessity for more careful monitoring of elderly or underweight patients treated with cytotoxic agents, including alkylating agents, antimetabolic agents, antineoplastic antibiotics, platinating agents, and plant-derived alkaloids. Our findings should facilitate the identification of drugs that may cause neutropenia and help healthcare professionals manage drug-induced adverse effects in their patients. Further verification and investigation of the underlying mechanisms in future studies are expected to extensively contribute to the understanding of the CIN risk revealed in our study.

## Figures and Tables

**Table 1:** Patient characteristics.

Characteristic	CIN	Non-CIN	*p* Value
Age (years)			
Median [range]	61 (0–103)	58 (0–110)	
≥65 years	13,397 (41.4%)	731,626 (35.9%)	<0.0001
<65 years	18,994 (58.6%)	1,309,160 (64.1%)	
Sex, No			
Female	16,751 (51.7%)	1,235,937 (60.6%)	<0.0001
Male	15,640 (48.3%)	804,849 (39.4%)	
Weight (kg)			
Median [range]	68.0 (0.0–150.0)	73.0 (0.0–150.0)	<0.0001
Antineoplastic agents			
Yes	19,720 (60.9%)	260,981 (12.8%)	<0.0001
No	12,671 (39.1%)	1,779,805 (87.2%)	
ATC classification			
Alkylating agent	1890 (4.8%)	10,279 (0.3%)	<0.0001
Antimetabolic agent	4078 (10.4%)	34,168 (1.2%)	
Antineoplastic agent	1054 (2.7%)	5843 (0.2%)	
Monoclonal antibody	3534 (9.0%)	59,542 (2.0%)	
Platinating agent	2111 (5.4%)	17,077 (0.6%)	
Protein kinase agent	2430 (6.2%)	72,130 (2.4%)	
Plant-derived alkaloids	2347 (6.0%)	27,673 (0.9%)	

ATC, Anatomical Therapeutic Chemistry. CIN, Chemotherapy-induced Neutropenia.

**Table 2:** Odds ratio related with CIN.

Variables	Category	Univariate Analysis	
		Odds Ratio (95% CI)	*p* Value
Age (years)	≥65 vs. <65	1.394 (1.365–1.424)	<0.0001
Sex	Female vs. male	1.434 (1.403–1.466)	<0.0001
Chemotherapeutic agents	Yes vs. No	10.614 (10.375–10.857)	<0.0001

**Table 3:** Principal component load related to CIN.

Adverse Event	First Component	Second Component
Granulocytopenia	0.594	0.670
Idiopathic neutropenia	0.167	0.383
Agranulocytosis	0.801	0.242
Neutropenia	0.904	0.0118
Febrile neutropenia	0.913	−0.141
Neutropenic colitis	0.794	−0.179
Neutropenic infection	0.802	−0.187
Neutropenic sepsis	0.853	−0.288

**Table 4:** Cross-tabulation and calculation formula for RORs of CIN.

Report Type	CIN	Non-CIN
Reports with the suspected medicine	a	c
All other reports	b	d

ROR = (a/b)/(c/d) = ad/bc.

## Data Availability

Data is contained within the article.
